# Pannexin1 deletion in lymphatic endothelium affects lymphatic function in a sex‐dependent manner

**DOI:** 10.14814/phy2.16170

**Published:** 2024-07-31

**Authors:** Avigail Ehrlich, Graziano Pelli, Robert Pick, Linda Clochard, Filippo Molica, Brenda R. Kwak

**Affiliations:** ^1^ Department of Pathology and Immunology (PATIM) University of Geneva Geneva Switzerland; ^2^ Geneva Center for Inflammation Research (GCIR), Faculty of Medicine University of Geneva Geneva Switzerland

**Keywords:** lymphatic endothelial cells, lymphatic function, pannexin1, sex differences

## Abstract

The lymphatic network of capillaries and collecting vessels ensures tissue fluid homeostasis, absorption of dietary fats and trafficking of immune cells. Pannexin1 (Panx1) channels allow for the passage of ions and small metabolites between the cytosol and extracellular environment. Panx1 channels regulate the pathophysiological function of several tissues in a sex‐dependent manner. Here, we studied the role of Panx1 in lymphatic function, and potential sex‐dependent differences therein, in *Prox1‐CreER* ^*T2*^ *Panx1* ^*fl/fl*^ and *Panx1* ^*fl/fl*^ control mice. Panx1 expression was higher in lymphatic endothelial cells (LECs) of male mice. Lymphatic vessel morphology was not affected in *Prox1‐CreER* ^*T2*^ *Panx1* ^*fl/fl*^ male and female mice. Lymphatic drainage was decreased by 25% in male *Prox1‐CreER* ^*T2*^ *Panx1* ^*fl/fl*^ mice, but was similar in females of both genotypes. Accordingly, only male *Prox1‐CreER* ^*T2*^ *Panx1* ^*fl/fl*^ mice exhibited tail swelling, pointing to interstitial fluid accumulation in males upon Panx1 deletion in LECs. Moreover, serum triglyceride and free fatty acid levels raised less in *Prox1‐CreER* ^*T2*^ *Panx1* ^*fl/fl*^ mice of both sexes in an oral lipid tolerance test. Finally, the percentage of migratory dendritic cells arriving in draining lymph nodes was increased in *Prox1‐CreER* ^*T2*^ *Panx1* ^*fl/fl*^ female mice, but was comparable between male mice of both genotypes. Our results point to a LEC‐specific role for Panx1 in the functions of the lymphatic system.

## 1 INTRODUCTION

The lymphatic vasculature forms a unidirectional transport pathway ensuring tissue fluid homeostasis (Petrova & Koh, 2020). Lymphatic capillaries start blind‐ended in tissues, possess an incomplete basement membrane and consist of a single layer of lymphatic endothelial cells (LECs) with overlapping borders and linked to each other by discontinuous, button‐like junctions enabling the absorption of the lymph from the interstitium and its drainage into collecting lymphatic vessels (Zhang et al., 2020). The latter are composed of a series of lymphangions, covered with a complete basement membrane and smooth muscle cells, separated by intraluminal valves (Wiig & Swartz, 2012). LECs in collecting lymphatic vessels have an elongated shape and continuous zipper‐like junctions (Zhang et al., 2020). Smooth muscle cell contractions and valves contribute to the unidirectional lymph flow towards the lymph nodes (LNs) (Scallan et al., 2016). Draining LNs receive immune cells, such as dendritic cells (DCs), from inflamed tissues. Chemokine gradients regulate the entrance of DCs into and their exit from lymphatic vessels, and DCs actively interact with LECs during their journey (Collado‐Diaz et al., 2022). Subsequently, the lymph drains into the thoracic duct or right lymphatic trunk to return to the bloodstream (Aspelund et al., 2016).

LECs represent a distinct endothelial cell lineage, and lymphatic vessels are often distinguished from blood vessels based on their expression of the lymphatic transcription factor prospero homeobox‐1 (Prox1) or the lymphatic vessel hyaluronan receptor‐1 (LYVE‐1) (Petrova & Koh, 2020). Lymphangiogenesis involves vascular endothelial growth factor C (VEGF‐C) to induce LEC proliferation (Karaman et al., 2018) and may require the presence of a membrane channel protein called Pannexin1 (Panx1) (Boucher et al., 2018).

Panx1 is part of a glycoprotein family, along with Panx2 and Panx3. Panx1 proteins form heptameric membrane channels, enabling the passage of small signaling molecules, including ATP, between the intra‐ and extra‐cellular space (Ehrlich et al., 2021). While Panx2 and Panx3 display distinct expression patterns, Panx1 is ubiquitously expressed, including in LECs (Boucher et al., 2018; Molica et al., 2017).

Ubiquitous deletion of Panx1 impaired lymphatic function resulting in reduced serum triglyceride (TG) levels in atherosclerosis‐prone apolipoprotein E‐deficient (*Apoe* ^*−/−*^) mice (Molica et al., 2017). Whether the lymphatic impairment in Panx1‐deficient mice resulted from altered LEC function or from secondary effects of Panx1 deletion in other cell types was however not investigated in this study. Here, we investigated the effects of a specific deletion of Panx1 in lymphatic endothelium on the functioning of lymphatic vessels using *Prox1‐CreER* ^*T2*^ *Panx1* ^*fl/fl*^ and *Panx1* ^*fl/fl*^ control mice. Interestingly, Panx1 may play a different pathophysiological role in males and in females. For instance, osteocytic Panx1 deletion increased bone and muscle mass only in female mice (Aguilar‐Perez et al., 2019) and ubiquitous Panx1 deletion decreased cerebral infarct size in female but not in male mice (Freitas‐Andrade et al., 2017). Therefore, we investigated potential sex differences in the role of Panx1 channels in lymphatic endothelium as well.

## 2 MATERIALS AND METHODS

### 2.1 Animals

Animal experimentation conformed to the Guide for the Care and Use of Laboratory Animals published by the US National Institutes of Health (NIH Publication No. 85–23, revised 1966) and was approved by the Swiss cantonal and federal veterinary authorities. *Prox1‐CreER* ^*T2*^ mice were kindly provided by Prof. Taija Makinen (Bazigou et al., 2011), and were bred with *Panx1* ^*fl/fl*^ mice (Molica et al., 2017) to obtain a *Prox1CreER* ^*T2*^ *Panx1* ^*fl/fl*^ mouse line; hereafter called *Panx1* ^*LECdel*^. *Prox1‐CreER* ^*T2*^ *Panx1* ^*fl/fl*^ mice were crossed with *Panx1* ^*fl/fl*^ mice, and 6 weeks‐old transgenic and control littermates were intraperitoneally (i.p.) injected with 1 mg tamoxifen (Sigma, cat no. T5648‐1G) in kolliphor oil (Sigma, cat no. C5135) twice per day for 4 days to induce Cre‐recombinase activity and subsequent Panx1 deletion in LECs. Mice were used for experiments 2 weeks after the last injection. Mice were killed under general anesthesia induced by i.p. injection with ketarom (10 mg/kg xylazine mixed with 100 mg/kg ketamine). All experiments were performed using both male and female mice.

### 2.2 Immunofluorescent staining

Immunostaining on cryosections (5 μm) of ears, jejunum and LN was performed using antibodies against Panx1 (HRB459; 1/100, Geneva Antibody Facility) (Rusiecka et al., 2019) and LYVE1 (1/500; Angiobio, cat no. AN11‐034). In brief, after fixation with 1% PFA (15 min), permeabilization with 0.2% TritonX‐100 (60 min), incubation with 0.5 M NH_4_Cl (15 min) and blocking with 10% normal goat serum (45 min), sections were incubated overnight with primary antibodies at 4°C. Incubation with the corresponding secondary antibodies Alexa fluor 488 (Invitrogen, cat no. A11013) and Alexa fluor 568 (Invitrogen, cat no. A11036) was performed at room temperature (RT) for 2 h. Nuclei were stained with 4′,6‐diamidino‐2‐phenylindole (DAPI) for 10 min (1/20000; Invitrogen, cat no. D1306). Slides were mounted with Vectashield mounting medium (Vector Laboratories, cat No. H‐1000) and analyzed using a Zeiss LSM800 confocal microscope. Orthogonal views were obtained using ImageJ (Fiji) software. Immunofluorescent staining on liver cryosections (5 μm) was performed as described above using antibodies against Panx1 (HRB459; 1/100, Geneva Antibody Facility). Images were captured with an Axiocam Fluo microscope (Zeiss). Quantification on seven images for each mouse was performed using Fiji software.

The nonfixed ears of *Panx1* ^*fl/fl*^ and *Panx1* ^*LECdel*^ mice were opened and separated into dorsal and ventral sheets. Cartilage‐free ventral ear sheets were rinsed with phosphate buffered saline (PBS) in 24‐well plates and incubated for 30 min with conjugated LYVE‐1 antibody (1/100; Invitrogen, cat no. 53–0443‐82). The samples were fixed with 4% PFA during 45 min and then mounted on a glass slide with a drop of PBS. Whole‐mount images were obtained using a Zeiss Axio Examiner.Z1 confocal spinning disk microscope. The Z‐stacks were converted to a single plane with ImageJ using the Z Project plugin. The LYVE‐1 positive area and vessel width were quantified using Fiji software.

### 2.3 Lymphatic endothelial cell extraction and cell sorting

LEC culture was performed as previously described (Garnier et al., 2022). Briefly, LNs of the skin were dissected, cleaned, pooled and incubated in a digestion mix composed of RPMI‐1640 (Gibco), dispase (0.8 mg/mL; Gibco, cat no. 17105–041), collagenase‐P (0.2 mg/mL; Roche, cat no. 11249002001), and DNase‐I (0.1 mg/mL; Roche, cat no. 10104159001). After incubation at 37°C, LNs were gently mixed every 10 min until total digestion of the tissues. Samples were then centrifuged and filtered through a 70 μm cell strainer. Cells were seeded in 6‐well plates (precoated with human fibronectin (10 μg/mL; Merck, cat no. FC010) and PureCol (10 μg/mL; Cellsystems, cat no. 5005‐100ML) for 30 min at 37°C) at a concentration of 5 × 10^6^ cells/well. Every 2 days, the cells were rinsed and the culture medium renewed. After 7 days, cells were detached with Accutase (Gibco, cat no. A1110501) for 5 min at 37°C. Cells were stained with CD31 (1/200; BD Pharmingen, cat no. 553372), GP38 (1/200; Invitrogen, cat no. 25–5381‐82) and CD45 (1/400; Invitrogen, cat no. 12–0451‐83). The LECs were sorted using flow cytometry (Biorad S3; Beckman Coulter). The dead cells and doublets were first discarded from the total cell population to select thereafter LECs as CD45^neg^, GP38^+^ and CD31^+^ cells.

### 2.4 Real‐time PCR

Total RNA from LECs was extracted using the RNAqueous‐Micro Kit (Thermo Fisher, cat no. AM1931) according to the manufacturer's instructions. cDNA was obtained using the Quantitect Reverse Transcription kit (Qiagen, cat no. 205311) and real‐time PCR (RT‐PCR) was carried out with the QuantStudio™ 6 Pro (Thermo Fisher) using the TaqMan Fast Universal master mix (Applied Biosystem, cat no. 4352046). Mouse Panx1 (Thermo Fisher, cat no. Mm00450900_m1) or Glyceraldehyde‐3‐phosphate dehydrogenase (GAPDH) (Thermo Fisher, cat no. Mm99999915_g1) primers and probes were purchased from Applied Biosystems. Measurements were performed in triplicates. Panx1 mRNA expression was normalized to GAPDH expression.

### 2.5 Western blot

Proteins were extracted from LECs or skeletal muscle using a RIPA lysis buffer as described elsewhere (Morel et al., 2014). Proteins were quantified with a colorimetric BCA protein assay kit (Thermo Fisher, cat no. 23235) according to manufacturer's instructions. Thirty micrograms of total protein were loaded on 10% SDS‐polyacrylamide gels, electrophoresed and electrotransferred onto PVDF transfer membranes (Immobilon Millipore). Transfer efficiency was checked with Ponceau S staining (Sigma, cat no. P7170‐L) or Revert™ 700 total protein stain (LICORbio, cat no. 926–11015) according to the manufacturer's instructions. After rinsing, membranes were subsequently blocked for 2 h with 5% milk in PBS‐Tween, and incubated overnight at 4°C with Panx1 (1/500; Cell Signaling, cat no. 91137S) or GAPDH (1/30000; Millipore, cat no. MAB374) primary antibodies. After 1 h incubation at RT with the respective secondary goat anti mouse horse radish peroxidase (1/5000; Jackson Immuno Research, cat no. 115–0035‐146) and goat anti rabbit horse radish peroxidase (1/5000; Jackson Immuno Research, cat no. 111–0035‐003) antibodies, revelation was performed with an ECL detection kit (Millipore, cat no. WBKLS0500) using the Fuji LAS3000 (Fujifilm) and ImageQuant LAS4000 software. Quantification was performed using ImageJ software or Empira Studio (LICORbio).

### 2.6 Lymphatic drainage and tail diameter measurement

Drainage of interstitial fluids was measured after injecting 5% Evans blue (5 μL, SIGMA, cat no. E2129‐50G) with a micro‐syringe in the left footpad of anesthetized *Panx1* ^*fl/fl*^ or *Panx1* ^*LECdel*^ mice (Meens et al., 2017). After 15 min, blood was collected by puncturing the left heart ventricle and centrifuged for 15 min at 5000 rpm (4°C). Sera were incubated overnight with formamide (500 μL per 200 μL serum; Sigma, cat no. F9037) at 55°C. Thereafter, the amount of Evans blue present in each serum sample was measured by fluorescence reading using a SpectraMax Paradigm Multi‐Mode Microplate reader (excitation: 620 nm; emission 680 nm; Molecular Devices). In addition, tail diameters of anesthetized *Panx1* ^*fl/fl*^ and *Panx1* ^*LECdel*^ mice were measured using a digital caliper at 1 cm from the basis and at 4 cm from the tip, and were normalized to body weight. Finally, tail samples were collected at 1 cm from the tip, snap frozen in OCT compound, cryosectioned (5 μm) and stained with Hematoxylin/Eosin for histological examination.

### 2.7 Oral lipid tolerance test

The uptake of long‐chain fatty acids was quantified as described before (Meens et al., 2017). *Panx1* ^*fl/fl*^ and *Panx1* ^*LECdel*^ mice were subjected to a 6 h fasting period. Immediately thereafter, the mice received a bolus of olive oil (10 μL/g body weight; Sigma, cat no. 1478265) by oral administration. Blood was sampled by submandibular puncture before (T = 0), 2 and 3 h after olive oil administration. TG and free fatty acid (FFA) concentrations were measured in mouse sera using a Cobas C111 analyzer (Roche Diagnostics).

### 2.8 Skin hypersensitivity assay

A contact hypersensitivity assay was performed on *Panx1* ^*LECdel*^ and *Panx1* ^*fl/fl*^ mice, and DC migration to draining LNs was evaluated using previously established methods (Molica et al., 2017). Briefly, a mixture of 1:1 acetone (Acros Organics, cat no. 176800025) and dibutyl phthalate (Sigma, cat no. 524980–25 mL) was applied to the skin of the right flank of the mice for 24 h. Thereafter, draining LNs were isolated, grinded and digested at 37°C in Hank's balanced salt solution (HBSS) containing collagenase‐D (1 mg/mL; Roche, cat no. 11088882001) and DNase‐I (10 μg/mL; Roche, cat no. 10104159001) for 40 min. The reaction was stopped by adding a 10% fetal calf serum solution containing 0.5 mM EDTA and 0.25% bovine serum albumin (FACS buffer). Samples were then filtered using a 70 μm cell strainer, centrifuged for 5 min at 1500 rpm and resuspended in FACS buffer with CD45 (1/200; BioLegends, cat no. 103134), CD11c (1/400; BioLegends, cat no. 117333) and MHC‐II (1/300; BioLegends, cat no. 107617) antibodies. The total number of DCs was counted by flow cytometry as CD45^+^, CD11c^+^, and MHCII^+^ cells. The percentage of CD11c^int^MHCII^hi^ DCs determined the newly arrived DCs in LNs. LNs from the left flank skin served as controls.

### 2.9 Statistical analysis

Graphpad Prism 9 was used for statistical analysis. Results were presented as mean ± SD. Two‐group comparisons were performed using unpaired one‐tailed or two‐tailed Student's *t‐*tests. Differences with a *p* ≤ 0.05 are considered as significant; exact significant *p* values are given on graphs.

## 3 RESULTS

### 3.1 Panx1 is differentially expressed in LECs of male and female mice

**FIGURE 1:**
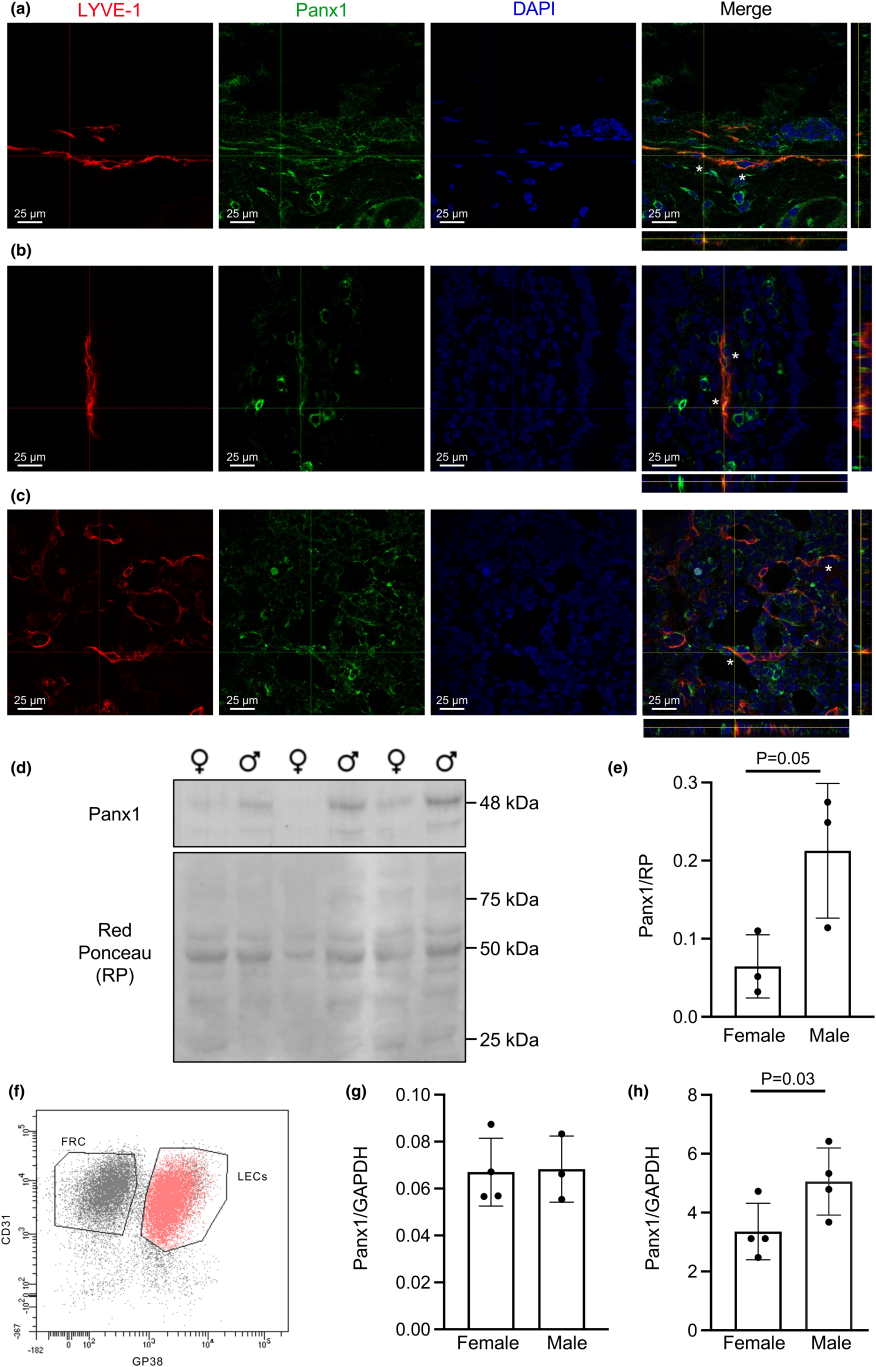
Panx1 expression is higher in LECs of male mice. Orthogonal views of co‐immunofluorescent staining for LYVE‐1 (red) and Panx1 (green) on lymphatic capillaries in ears (a), intestinal villi (b) and LNs (c). Nuclei were stained with DAPI (blue). Asterisks indicate Panx1 and LYVE‐1 colocalization in the merged images. (d) Representative Western blot of Panx1 (41–48 kDa) in skeletal muscle of female and male mice. Red ponceau was used as a loading control. (e) As expected, Panx1 protein expression is higher in skeletal muscle of male mice (one‐tailed Student's *t*‐test; *N* = 3). (f) Representative image of the gating strategy used to obtain a pure population of LECs (red). (g) Panx1 mRNA expression in LECs extracted from LNs is equal in female (*N* = 4) and male (N = 3) mice. (h) Panx1 protein expression is higher in LECs of male mice (one‐tailed Student's *t*‐test; *N* = 4).

Panx1 expression was first examined by immunofluorescent staining in lymphatic capillaries of three different territories in control mice. Panx1 was found in lymphatic capillaries of the ears, where it colocalized with the LEC marker LYVE‐1 (Figure 1a). Additionally, we found a colocalization between Panx1 and LYVE‐1 in lacteals of intestinal villi (Figure 1b) and in capillaries of LNs (Figure 1c).

We then wanted to investigate if the level of Panx1 expression was influenced by the sex of the mice. As a control, we first performed Western blotting on skeletal muscle (Figure 1d), a tissue where sex differences have previously been reported (Aguilar‐Perez et al., 2019). As expected, Panx1 expression was increased in skeletal muscle of male mice compared to female mice (Figure 1e).

Next, we examined if lymphatic endothelium of male mice also expressed more Panx1, and we performed real‐time PCR and Western blotting on a pure population of LECs. To reduce the number of animals used for these experiments, cells were isolated from LNs of male and female wild‐type mice, cultured for 1 week after which LECs were selected by FACS as CD45^neg^, GP38^+^ and CD31^+^ cells, and RNA and protein extractions prepared (Figure 1f). To our surprise, Panx1 mRNA expression levels were similar in LECs obtained from mice of both sexes (Figure 1g) while Panx1 protein expression was higher in LECs from male mice (Figure 1h). Together, these results demonstrate sex‐dependent alterations in the level of Panx1 expression in LECs, which seem not to result from transcriptional but rather from translational differences or from differences in protein degradation between both sexes.

### 3.2 Panx1 deletion does not modify lymphatic vessel morphology in ears

**FIGURE 2:**
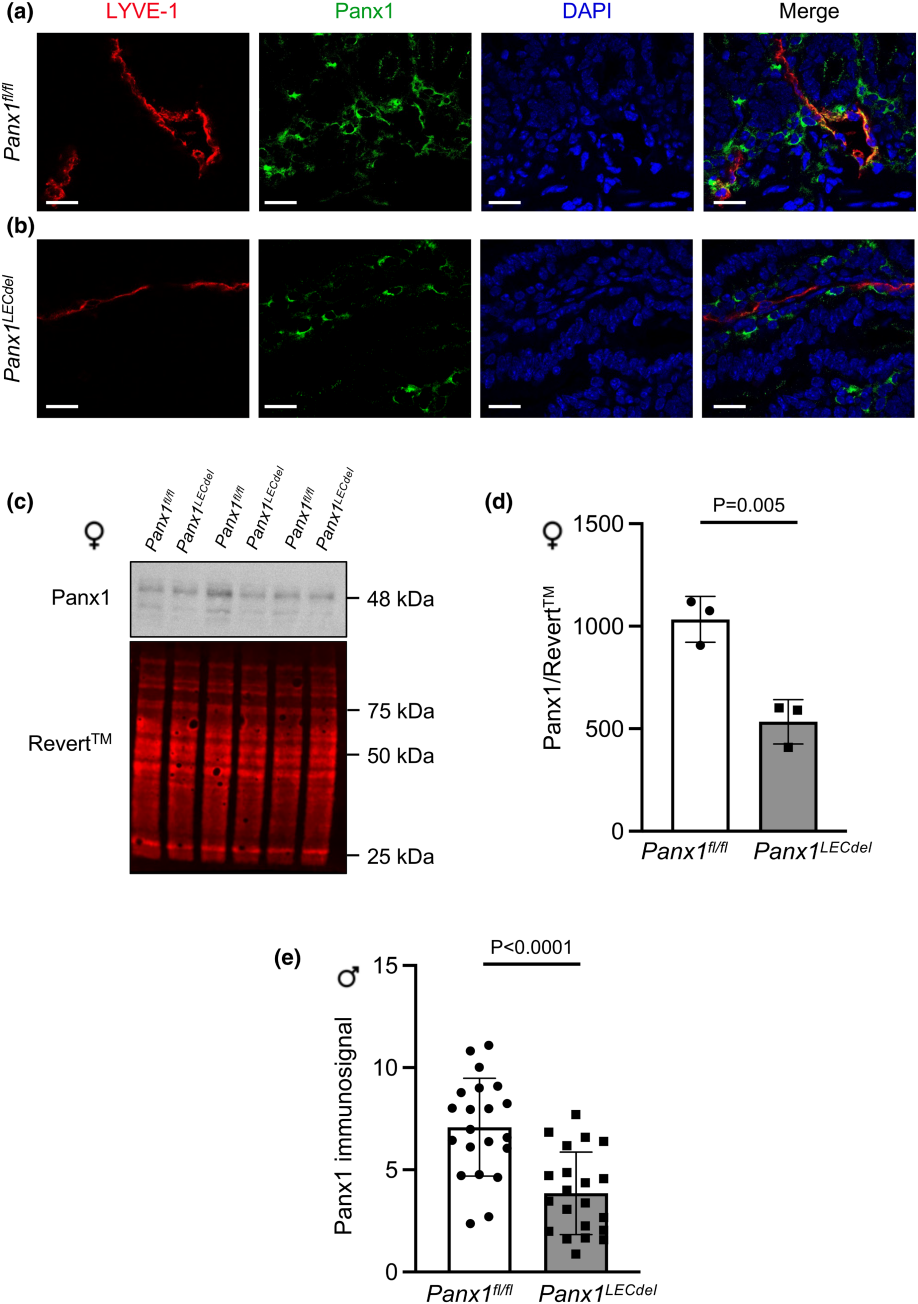
Panx1 deletion in *Panx1* ^*LECdel*^ and control mice. Verification of tamoxifen‐induced deletion of Panx1 in intestinal villi of control *Panx1* ^*fl/fl*^ (a) and *Panx1* ^*LECdel*^ mice (b) stained for LYVE‐1 (red) and Panx1 (green). Nuclei were stained with DAPI (blue). (c) Representative Western blot of Panx1 (41–48 kDa) in whole liver lysates of female *Panx1* ^*fl/fl*^ and *Panx1* ^*LECdel*^ mice. Revert™ 700 total protein stain was used as a loading control. (d) Panx1 protein expression is reduced in whole liver lysates from female *Panx1* ^*LECdel*^ mice compared with female *Panx1* ^*fl/fl*^ whole liver lysates (two‐tailed Student's *t*‐test; N = 3). (e) Panx1 immunosignal is reduced in liver of male *Panx1* ^*LECdel*^ mice compared with male *Panx1* ^*fl/fl*^ livers (two‐tailed Student's *t*‐test; *N* = 3, *n* = 21).

To verify the deletion of Panx1 from LECs after induction of Cre‐recombinase activity in *Panx1* ^*LECdel*^ mice, we performed co‐immunofluorescent staining for Panx1 and LYVE‐1 on cryosections of jejunum. Whereas Panx1 colocalized with LYVE‐1 in the lymphatic vessels of *Panx1* ^*fl/fl*^ mice, Panx1 was absent from the lymphatic vessels of *Panx1* ^*LECdel*^ mice 2 weeks after tamoxifen injection (Figure 2a,b). However, Panx1 immunosignal was observed at this time point in cells surrounding the lymphatic vessel (Figure 2b), illustrating the LEC specificity of the deletion. These results confirm Cre‐recombinase‐induced deletion of Panx1 in LECs at 2 weeks after tamoxifen treatment.

Of note, Prox1 is considered an early marker of hepatoblasts and its expression has also been described in adult hepatocytes, in particular after injury and during liver regeneration (Dudas et al., 2006). Therefore, we performed Western blotting of livers from female *Panx1* ^*LECdel*^or *Panx1* ^*fl/fl*^ mice. As compared to *Panx1* ^*fl/fl*^ mice, Panx1 expression was markedly decreased (by 50%) in whole liver lysates from *Panx1* ^*LECdel*^ mice at 2 weeks after tamoxifen treatment (Figure 2c,d). As Panx1 is ubiquitously expressed, the remaining Panx1 signal in the whole liver lysates of *Panx1* ^*LECdel*^ mice can likely be ascribed to the non‐hepatocyte cell fraction. A similar decrease in Panx1 levels was observed by immunofluorescent staining of liver sections from male *Panx1* ^*LECdel*^and *Panx1* ^*fl/fl*^ mice (Figure 2e).

**FIGURE 3:**
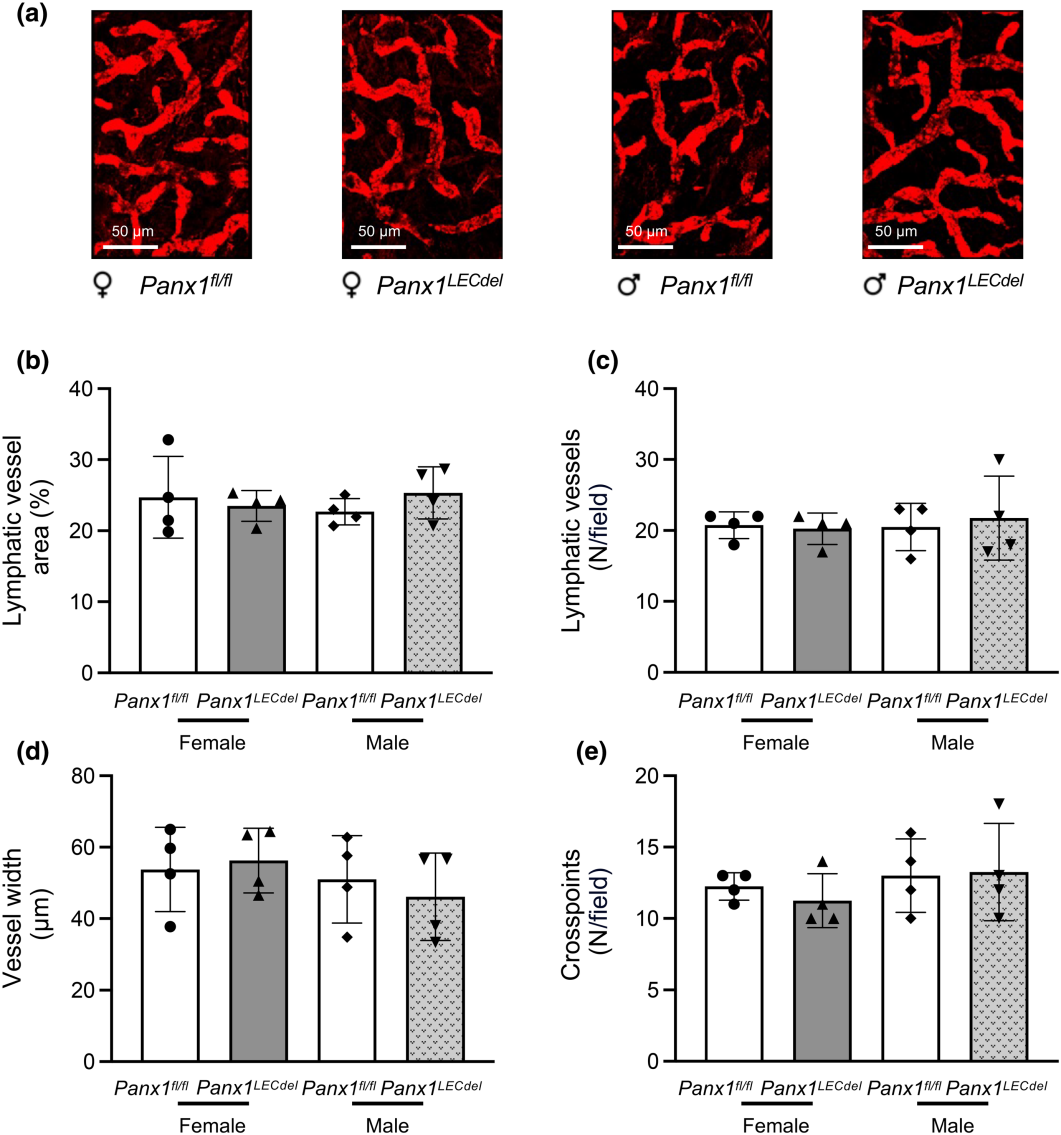
Lymphatic vessel morphology is similar in ears of male and female *Panx1* ^*LECdel*^ and control mice. (a–e) Whole‐mount staining for LYVE‐1 (red) on ears of control *Panx1* ^*fl/fl*^ (white bars) and *Panx1* ^*LECdel*^ female (gray bars) and male (patterned gray bars) mice. The area covered by lymphatic vessels (b), vessel number (c), vessel width (d) and the numbers of cross‐points (e) were quantified. (*N* = 4).

As Panx1 was required for in vitro lymphangiogenesis (Boucher et al., 2018), we checked whether Panx1 deletion from LECs would affect the morphology of the lymphatic network using LYVE‐1 immunostaining and spinning disk microscopy on ears of both male and female *Panx1* ^*LECdel*^or *Panx1* ^*fl/fl*^ control mice (Figure 3a). Neither the genotype nor the sex of the mice affected the lymphatic vessel density, the number of lymphatic vessels per field, the vessel width or the number of crosspoints per field (Figure 3b–e). Thus, morphological features of the ear lymphatic vasculature are not affected by Panx1 deletion in LECs of both male and female mice.

### 3.3 Panx1 deletion in LECs reduces intestinal fat absorption in male and female mice

**FIGURE 4:**
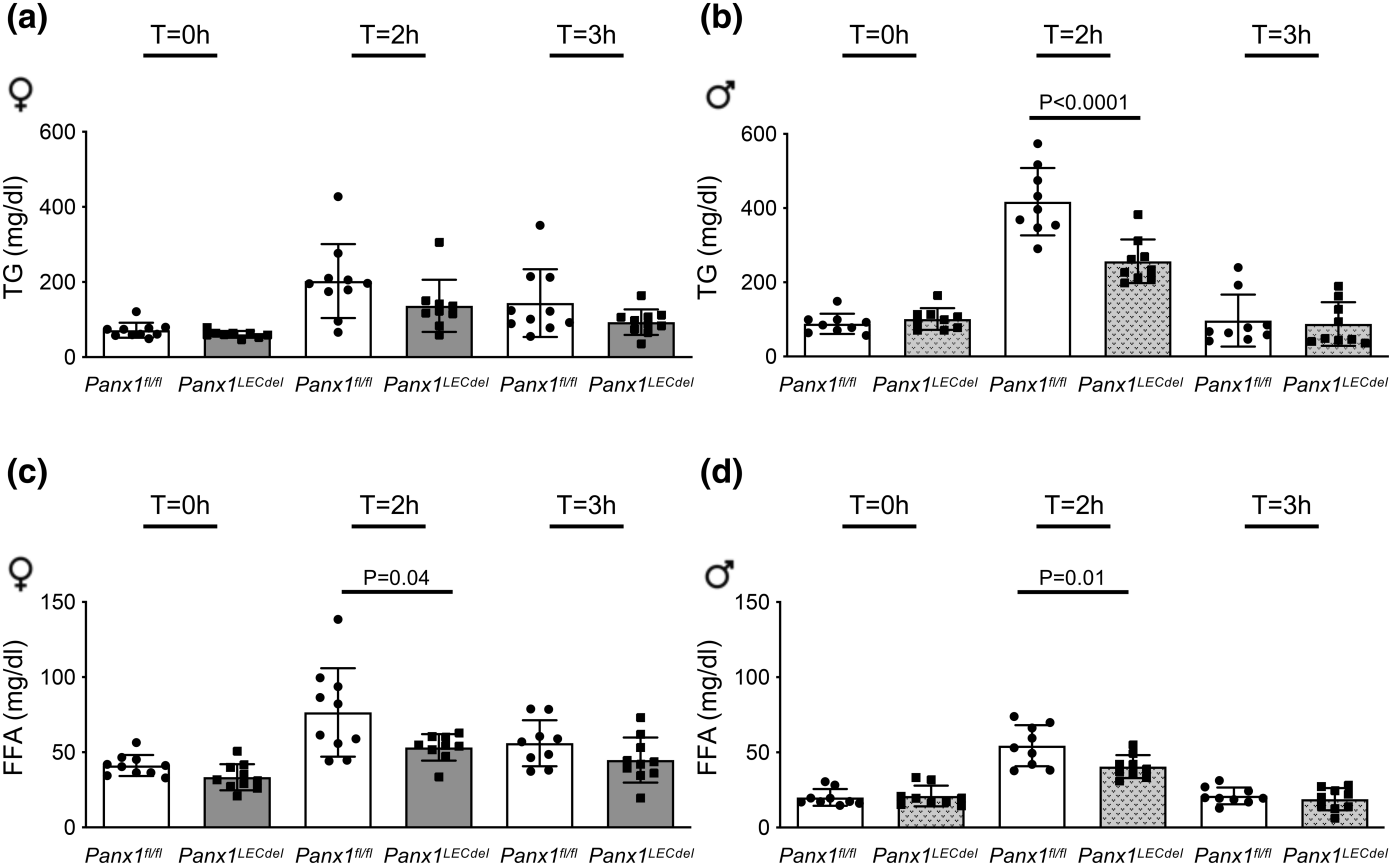
LEC‐specific Panx1 deficiency reduces dietary fat absorption in both male and female mice. TG and FFA concentrations before (T = 0 h), at 2 h and 3 h after oral administration of olive oil to control *Panx1* ^*fl/fl*^ (white bars) and *Panx1* ^*LECdel*^ (gray bars) female (a and c) and male mice (b and d). (*N* = 9). Two‐tailed Student's *t*‐test.

Next, we investigated the effects of Panx1 deletion in LECs on the various lymphatic functions. Lacteals are important for the uptake of dietary fat and fat‐soluble vitamins from the small intestine. To investigate the effect of Panx1 deletion in LECs on the uptake of long‐chain fatty acids we subjected *Panx1* ^*fl/fl*^ and *Panx1* ^*LECdel*^ mice to an oral lipid tolerance test by administration of a bolus of olive oil and measured serum lipids 2 and 3 h later. Before the gavage (T = 0 h) the levels of TG were similar in mice of both genotypes and sexes (Figure 4a,b). The FFA levels before gavage were also not different between genotypes, however, in agreement with an earlier study (Christeff et al., 1994), FFA levels were higher in females than in male mice at T = 0 h (Figure 4c,d). Interestingly, the FFA level raised less at 2 h after the gavage in both male and female *Panx1* ^*LECdel*^ mice as compared to *Panx1* ^*fl/fl*^ control mice (Figure 4c,d). A similar tendency was observed for TG levels in male and female *Panx1* ^*LECdel*^ mice (Figure 4a,b), suggesting altogether impaired dietary fat absorption in mice with Panx1 deletion in LECs. Of note, TG and FFA levels were returned to basal values at 3 h after the gavage in mice of both genotypes and sexes. These results suggest a regulatory role of Panx1 channels in LECs in the absorption of dietary fat from intestines in both male and female mice.

### 3.4 Panx1 deficiency in LECs reduces lymphatic drainage in male mice

**FIGURE 5:**
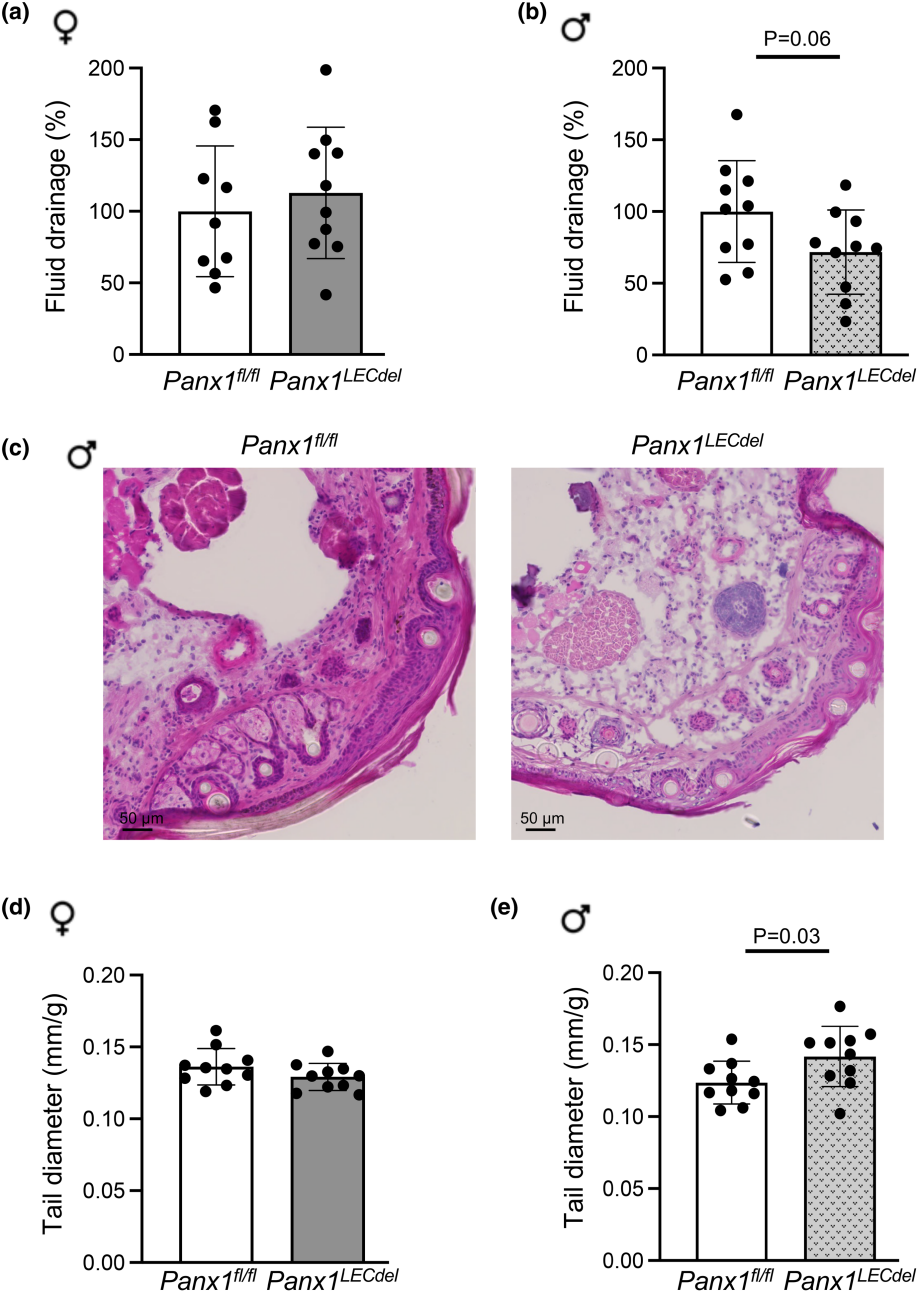
Lymphatic drainage is decreased in male *Panx1* ^*LECdel*^ mice. Interstitial fluid drainage was measured by quantification of Evans blue in the sera of *Panx1* ^*fl/fl*^ (white bars) and *Panx1* ^*LECdel*^ female (a) (gray bars; *N* = 10) and male mice (b) (patterned gray bars; *N* = 10). (c) Representative images of Hematoxylin/Eosin stained cryosections of tails from *Panx1* ^*fl/fl*^ and *Panx1* ^*LECdel*^ mice. Tail diameter in *Panx1* ^*fl/fl*^ and *Panx1* ^*LECdel*^ mice was measured at 1 cm from the tip in female (d) and male (e) mice (*N* = 10). Two‐tailed Student's *t*‐test.

We also investigated the effects of Panx1 deletion in LECs on lymphatic drainage. Similar to earlier studies (Meens et al., 2017; Molica et al., 2017), we injected Evans blue in the footpad of control *Panx1* ^*fl/fl*^or *Panx1* ^*LECdel*^ male and female mice and studied its propagation towards the blood circulation. Although lymphatic drainage was similar in females of both genotypes (Figure 5a), it was decreased by about 25% in male *Panx1* ^*LECdel*^ mice compared with *Panx1* ^*fl/fl*^ controls (Figure 5b), suggesting a sex‐specific impairment of lymphatic drainage upon Panx1 deletion in LECs.

Impaired lymphatic drainage of interstitial fluid may induce tail swelling in mice, as previously shown for *Apoe* ^*−/−*^ mice that are known to exhibit decreased lymphatic function (Lim et al., 2009). Hematoxylin/Eosin staining on cryosections of *Panx1* ^*LECdel*^ tails of male mice suggested increased interstitial fluid content as compared to male *Panx1* ^*fl/fl*^ tails (Figure 5c). Thus, we measured the tail diameter at two different locations, that is, 1 cm from the basis and 4 cm from the tip, in male and female *Panx1* ^*LECdel*^ or control *Panx1* ^*fl/fl*^ mice (Ehrlich et al., 2021). In agreement with the above results, only male *Panx1* ^*LECdel*^ mice exhibited larger tail diameters at both locations (see Figure 5e for the results at 1 cm from the tail tips), pointing to a tail fluid accumulation only in males but not in female mice (Figure 5d) upon Panx1 deletion from LECs. These results indicate a sex‐dependent role of Panx1 channels in the regulation of interstitial fluid drainage by lymphatic vessels.

### 3.5 Panx1 deletion in LECs affects DC migration in female mice

**FIGURE 6:**
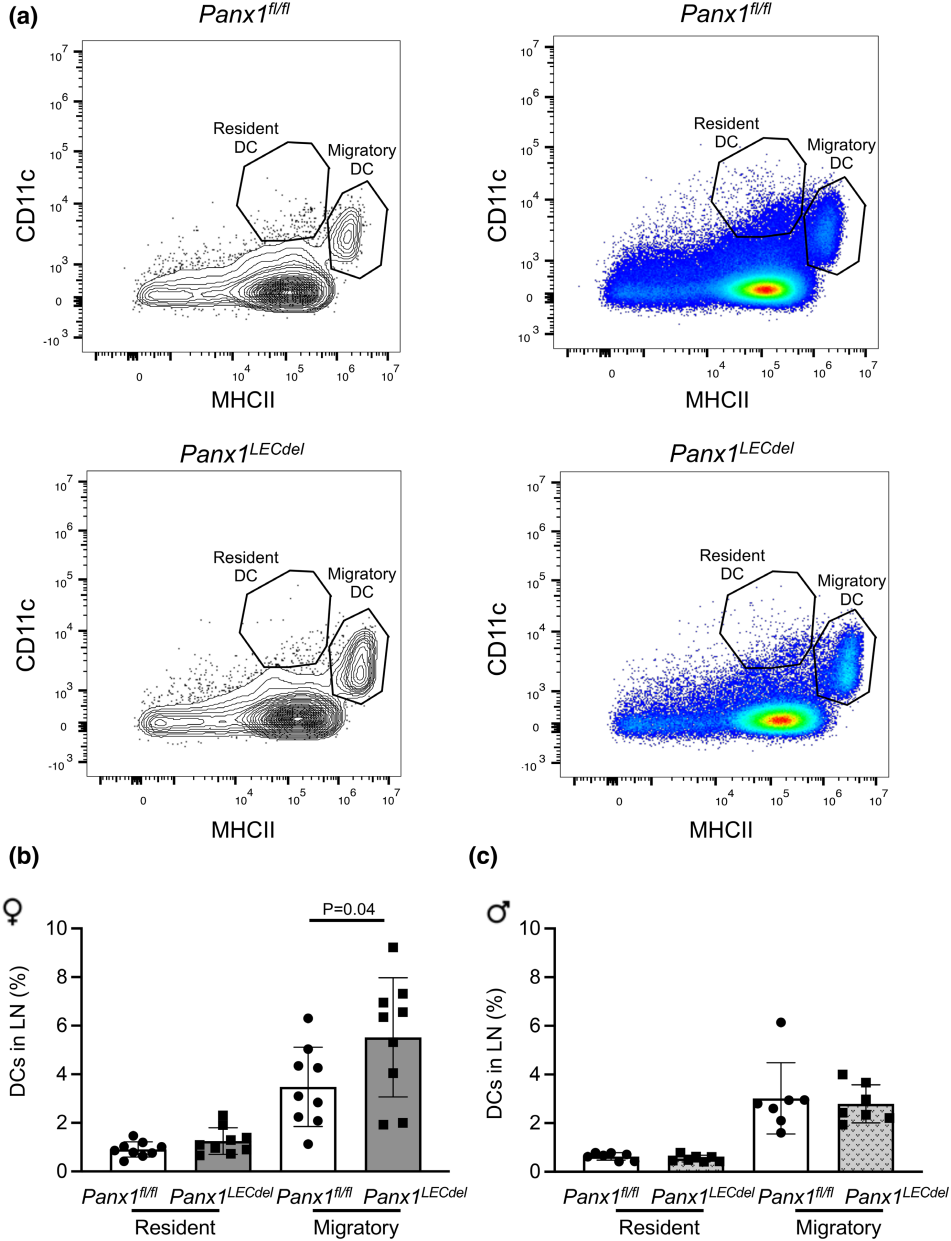
DC migration is increased in female mice upon Panx1 deletion. (a) Representative density (left panels) and total cell (right panels) images illustrating the gating strategy used to identify the populations of migratory DCs (CD11c^int^ MHC‐II^hi^) and resident DCs (CD11c^hi^ MHC‐II^int^) in LNs isolated from female *Panx1* ^*fl/fl*^ and *Panx1* ^*LECdel*^ mice. (b–c) Percentage of resident and migratory DCs, in draining LNs from *Panx1* ^*fl/fl*^ (white bars) and *Panx1* ^*LECdel*^ female (b) (gray bars; *N* = 9) and male (c) (patterned gray bars; *N* = 7) mice. Two‐tailed Student's *t*‐test.

Lymphatic vessels are also important players in the immune response. When DCs enter the lymphatic capillaries, they continuously interact with LECs and actively crawl towards collecting vessels to finally reach the draining LNs. To investigate whether Panx1 deletion affects this trafficking of DCs in the lymphatic vasculature, male and female *Panx1* ^*LECdel*^ and *Panx1* ^*fl/fl*^ mice were subjected to a skin contact hypersensitivity assay for 24 h after which draining LNs were isolated and the percentage of CD11c^int^MHCII^hi^ migratory DCs and resident CD11c^hi^MHCII^int^ DCs were determined by FACS (Figure 6a). As shown in Figure 6b,c, the percentage of resident DCs was similar between both genotypes in male mice. Interestingly, the percentage of migratory DCs into draining LNs was increased by about 2% in *Panx1* ^*LECdel*^ female mice in comparison with control *Panx1* ^*fl/fl*^ mice (Figure 6b). Despite the efficient induction of DC migration, no differences were observed between male mice of both genotypes (Figure 6c). Thus, Panx1 channels in LECs from female but not from male mice regulate DC trafficking towards draining LNs.

## 4 DISCUSSION

In this study we revealed differential sex‐dependent functional effects of Panx1 deletion in lymphatic endothelium. As described previously (Boucher et al., 2018; Molica et al., 2017), we found Panx1 expression in LECs in vitro (Figure 1g,h) but also in LECs of different functional territories in situ such as intestinal villi, ears and LNs (Figure 1a–c). Interestingly, the level of Panx1 protein expression in LECs was influenced by the sex of the mice, being higher in males. Transcriptional regulation of the Panx1 promoter by testosterone has been shown in the rat epididymis (Turmel et al., 2011), particularly in regions with high testosterone levels where the transcription factors ETV4 and CREB appeared to regulate the androgen‐mediated repression of Panx1 (Dufresne & Cyr, 2014; Turmel et al., 2011). Since we observed no sex‐dependent differences in Panx1 mRNA, this mechanism is unlikely to play a role in LECs, suggesting that the sex‐induced regulation of Panx1 protein occurred post‐transcriptionally. Sex hormones can directly or indirectly, through interactions with other regulatory proteins, affect the synthesis of specific proteins by regulating the translation of mRNAs. So far, no such effects have been described for Panx1. Alternatively, sex hormones may influence the turnover of proteins through various mechanisms, including protein stabilization, proteosomal degradation and autophagy. Interestingly, an augmentation in Panx1 expression in arterial endothelial cells has been recently shown to involve an oscillatory shear stress‐induced decrease in macro‐autophagy (Molica et al., 2022). Whether macro‐autophagy also plays a role in the sex‐dependent differences in Panx1 protein expression remains to be investigated. Finally, we also demonstrated increased Panx1 protein expression in the skeletal muscle of male wild‐type mice, a tissue in which the sex‐dependent functional roles of Panx1 have been previously described (Freeman et al., 2022). As sex‐dependent differences in Panx1 expression seems to extend to multiple, if not all, tissues, it was essential for this study to breed a mouse line with rather specific deletion of Panx1 in lymphatic endothelium.

We confirmed that tamoxifen‐induced deletion of Panx1 in LECs did not induce gross morphological differences in lymphatic vasculature between the genotypes for both males and females (Figure 3). Subsequently, we investigated the impact of Panx1 deletion in LECs on the three functions of the lymphatic system, i.e. drainage, absorption of dietary fat and the immune response (Ehrlich et al., 2021) and observed a differential sex‐dependent signature of Panx1 deletion on each of these functions. First, lymphatic drainage was impaired only in male mice upon Panx1 deletion from LECs, which is consistent with the results obtained in our previous study in atherosclerosis‐prone male mice with ubiquitous Panx1 deletion (Molica et al., 2017). Lymphatic drainage depends both on the pace by which interstitial fluid enters the lymphatic capillaries and the rate of propagation through the lymphatic collecting vessels. As the latter would affect the outcomes of the three lymphatic functions in a similar manner, a sex‐dimorphic effect of LEC‐specific Panx1 deletion on the bulk flow through the lymphatic collecting vessels is unlikely. Among other mechanisms, tight junctions ensure the integrity and permeability of lymphatic capillaries and may thus regulate the uptake of interstitial fluid. Sex differences exist in major components of tight junctions between LECs, for instance in Zona Occludens‐1 (ZO‐1). Indeed, it has been shown that ZO‐1 is downregulated by estradiol in females, which potentially leads to increased permeability and less efficient drainage (Zhou et al., 2017). Another study showed that blocking Panx1 channels prevented a cytokine‐dependent reduction of ZO‐1 and increase in epithelial permeability (Diezmos et al., 2018). Based on these studies, one may hypothesize that Panx1 deletion in LECs could prevent the downregulation of ZO‐1 caused by estradiol, thus explaining why lymphatic drainage remains similar between Panx1‐deficient and control female mice. Furthermore, since males have lower levels of estradiol (Iorga et al., 2017), they do not experience this ZO‐1 reduction (Zhou et al., 2017), resulting in a larger impact on drainage function when the Panx1 protein is deleted.

Secondly, we subjected *Panx1* ^*fl/fl*^ and *Panx1* ^*LECdel*^ mice to an oral lipid tolerance test to investigate the effect of Panx1 deletion in LECs on the uptake of long‐chain fatty acids. Thus, a defined dose of olive oil was administered by gavage and serum lipids were measured 2 and 3 h later. At these time points, the observed serum lipid levels will not only depend on lipid clearance from the gut but will also allude to liver function in repressing lipogenesis following gavage or taking up lipids from circulation once it is there. We found that serum TG and free fatty acid levels raised less in both in male and female mice upon Panx1 deletion from LECs. This is in agreement with our earlier study on male *Panx1* ^*−/−*^ *Apoe* ^*−/−*^ mice (Molica et al., 2017), and suggests a regulatory role of Panx1 in LECs in dietary fat absorption. CD36, also known as fatty acid translocase, is a fatty acid transporter expressed in LECs (Cifarelli et al., 2021; Glatz et al., 2022). Deletion of CD36 from LECs leads to leaky lymphatic vessels in the mesenteric region and a slower transport of absorbed lipids (Cifarelli et al., 2021). Interestingly, ubiquitous deletion of Panx1 in mice was associated to a > 2‐fold reduction in CD36 levels under control conditions as well as under conditions of acute and chronic liver disease (Willebrords et al., 2018). Although a female‐specific repression of hepatic CD36 in response to food deprivation has been found in rats, this was contrasted by a stimulatory effect in skeletal muscle (Cheung et al., 2007) and no sex‐specific differences of CD36 in LECs have been described so far. Nevertheless, these studies suggest a link between Panx1 and CD36 in the regulation of lipid metabolism and may explain a reduced uptake of dietary fat in male and female *Panx1* ^*LECdel*^ mice. It should be kept in mind however that Panx1 expression levels in the liver were also reduced by half in both male and female *Panx1* ^*LECdel*^ mice (Figure 2c–e), an effect which is likely due to tamoxifen‐induced deletion of Panx1 in Prox1‐expressing hepatocytes. The observed differences in the oral lipid tolerance test between *Panx1* ^*fl/fl*^ and *Panx1* ^*LECdel*^ mice may thus not only reflect differences lipid clearance from the gut but may also be influenced by potential differences in liver function. The 2 and 3 h time points chosen to measure serum lipid levels represent an important limitation of this study as an oral lipid tolerance test including earlier time points may better illustrate changes in lymphatic uptake rate.

Finally, DC migration to draining LNs was only enhanced in female mice upon Panx1 deletion from LECs (Figure 6b). DCs closely interact with LECs within capillaries and detach from these cells once they arrive in the collecting lymphatics, where lymph flow is enhanced (Collado‐Diaz et al., 2022). DCs are guided from the interstitium towards the lymphatic capillaries by CCL21 gradients, which are secreted by the LECs upon a Ca^2+^ influx stimulated by LEC‐DC contacts (Vaahtomeri et al., 2017). Similar to male mice with ubiquitous Panx1 deletion (Molica et al., 2017), the percentage of migratory DCs arriving in draining LNs was comparable between male *Panx1* ^*LECdel*^ and *Panx1* ^*fl/fl*^ mice (Figure 6c), suggesting that Panx1 is not directly involved in regulatory LEC‐DC interactions. It is increasingly recognized that estrogen signaling regulates crucial aspects of DC development and effector functions, both in mice and humans (Laffont et al., 2017). The female‐specific increase in DC response in the absence of Panx1 in LECs (Figure 6b) might result from a regulatory action of Panx1 on estrogen‐induced aspects of DC development and effector functions. Furthermore, although DC migration to draining LNs doubled in female *Panx1* ^*LECdel*^ mice, the pathophysiological impact of this 2% difference remains to be investigated in a disease setting.

Altogether, our results point to a role for Panx1 channels in the various functions of the lymphatic system. The different effects observed between sexes strongly suggest that the role of Panx1 channels might differ depending on the lymphatic function (cq. territory) studied.

## AUTHOR CONTRIBUTIONS

BRK conceived and designed research; AE, GP, RP, LC and FM performed experiments; AE and FM analyzed data; AE, FM and BRK interpreted results of experiments; AE prepared figures; AE, FM and BRK drafted the manuscript; all authors edited and revised manuscript; all authors approved the final version of manuscript.

## FUNDING INFORMATION

This study was supported by grants from the Swiss National Science Foundation (grant number 310030_182573 to BRK) and the Gabbiani fund (to AE).

## CONFLICT OF INTEREST STATEMENT

The authors declare no conflict of interest.

## Data Availability

The source data that support the findings are available in YARETA at https://doi.org/10.26037/yareta:govhyqqrkbesbmr5qaozanrghm.
